# Main drivers of health expenditure growth in China: a decomposition analysis

**DOI:** 10.1186/s12913-017-2119-1

**Published:** 2017-03-09

**Authors:** Tiemin Zhai, John Goss, Jinjing Li

**Affiliations:** 1 Centre for Research & Action in Public Health, Health Research Institute, University of Canberra, 2601 Canberra, ACT Australia; 2 Department of National Health Accounts and Policy Studies, China National Health Development Research Centre, Beijing, 100191 China; 3 Institute of Governance and Policy Analysis, University of Canberra, Canberra, 2601 ACT Australia

**Keywords:** Health expenditure, Drivers, Decomposition

## Abstract

**Background:**

In past two decades, health expenditure in China grew at a rate of 11.6% per year, which is much faster than the growth of the country’s economy (9.9% per year). As cost containment is a key aspect of China’s new health system reform agenda, this study aims to identify the main drivers of past growth so that cost containment policies are focussed in the right areas.

**Method:**

The analysis covered the period 1993–2012. To understand the drivers of past growth during this period, Das Gupta’s decomposition method was used to decompose the changes in health expenditure by disease into five main components that include population growth, population ageing, disease prevalence rate, expenditure per case of disease, and excess health price inflation. Demographic data on population size and age-composition were obtained from the Department of Economic and Social Affairs of the United Nations. Age- and disease- specific expenditure and prevalence rates by age and disease were extracted from China’s National Health Accounts studies and Global Burden of Disease 2013 studies of the *Institute for Health Metrics and Evaluation*, respectively.

**Results:**

Growth in health expenditure in China was mainly driven by a rapid increase in real expenditure per prevalent case, which contributed 8.4 percentage points of the 11.6% annual average growth. Excess health price inflation and population growth contributed 1.3 and 1.3% respectively. The effect of population ageing was relatively small, contributing 0.8% per year. However, reductions in disease prevalence rates reduced the growth rate by 0.3 percentage points.

**Conclusion:**

Future policy in optimising growth in health expenditure in China should address growth in expenditure per prevalent case. This is especially so for neoplasms, and for circulatory and respiratory disease. And a focus on effective interventions to reduce the prevalence of disease in the country will ensure that changing disease rates do not lead to a higher growth in future health expenditure; Measures should be taken to strengthen the capacity of health personnel in grass-roots facilities and to establish an effective referral system, so as to reduce the growth in expenditure per case of disease and to ensure that excess health price inflation does not grow out of control.

**Electronic supplementary material:**

The online version of this article (doi:10.1186/s12913-017-2119-1) contains supplementary material, which is available to authorized users.

## Objective

This study aims to identify each driver’s contribution to health expenditure growth in the past at the disease level to identify the main drivers of past growth. This enables the government to identify areas where future savings can be realised. The resulting estimates can also be useful for planning the future of the health care system in China.

## Background

Total health expenditure in China has grown considerably since economic reform started in 1978. Between 1978 and 2012 total health expenditure in China grew at a rate of 11.7% per annum, which is higher than that of gross domestic product (GDP) (9.9%), leading to an increase in the health expenditure share of GDP from 3.0 to 5.3% during the same period. The growth in health spending has been particularly rapid since the new round of health system reform was enacted in 2009 (13.1% annual average growth in the period 2009 to 2012). Since 2009 the income elasticity of health care had also increased by a factor of 1.4 [1]. The increasing cost of health care is a key focus of China’s new round of health system reform, with cost containment in public hospitals being a core aspect of the government agenda [2]. In this respect, understanding the main drivers of past health spending growth is important because, by identifying the main factors responsible for past growth, it enables the government to identify areas where future savings can be realised. The resulting estimates can also be useful for planning the future of the health care system in China. In the past, statistical methods were mainly used to identify the drivers of health expenditure growth. Regression methods, such as principal component regression, dynamic model and ridge regression analysis that have been used to analyse the drivers of aggregated total or regional health expenditure in China, have found that population ageing, income level and urbanisation were the main drivers [3–5]. However, their estimation of each factor’s contribution to health expenditure growth was not consistent, and some important drivers of health expenditure growth were not included, such as disease prevalence rates or health price inflation [3–5]. Previous studies did not identify the relative contribution of each factor to the growth of disease-specific expenditure. Fully allocating disease-specific expenditure growth into the main policy relevant drivers can offer detailed information for policy-making.

To our knowledge, this is the first study to comprehensively examine the policy-relevant drivers of health expenditure by disease in China, and identify the relative contribution of each factor to the growth of disease-specific expenditure in the past 20 years. The aim of this study is to decompose the main drivers of China’s health expenditure growth, with a special focus on the effects of changes in the disease prevalence rate, population size, population ageing, real expenditure per case of disease and excess health price inflation (EHPI).

## Methods

### Study design

Regression methods are commonly used to identify the main factors affecting health expenditure growth over time or across countries [6–10]. Specifically, the residual method is commonly used. This identifies a series of factors such as general inflation, population ageing, the spread of insurance and rising income that contribute to health expenditure growth; determines how much of the change they might account for; and attributes the residual to technological advances [11]. This method, however, may lead to an overestimate of the effect of technological progress if all of the residual is attributed to technological advances and other unobserved factors besides technology may be in the residual [12]. Residual methods use historical time series data to ensure a precise decomposition of growth of health expenditure [13].

Another approach can be used for decomposing the effect of different factors on health expenditure growth is the Das Gupta decomposition method [14, 15], which needs two time points of data only. This method is widely used in demographic and health studies [16, 17]. Das Gupta’s decomposition method decomposes the difference between two quantities (rates, means, proportions, ratios, or absolute values, etc.) into additive components (Additional file 1). This method develops several counterfactual scenarios and calculates the effect of each factor on changes from the present level assuming that all other factors, except the factor under consideration, remains the same during the study period. This method gives results that are independent of the order in which the factors are considered. In addition, instead of having a separate interaction component, Das Gupta’s decomposition method distributes interaction effects in proportion to the strength of each of the main effects. This distribution does not change conclusions about the relative importance of the factors. It only simplifies the picture [15]. In this study, we developed a model based on Das Gupta’s decomposition method to decompose the main drivers of health expenditure growth in China.

### The model

The health expenditure decomposed in this study is current health expenditure, which means capital formation is not included. Factors influencing health spending include demand-side factors, such as ageing and the health status of a population, income growth, and consumer behaviour; supply-side drivers, such as technological progress and changes in treatment practices, productivity, and health prices; and regulatory factors, such as institutional characteristics of health systems and their financing [18].

Considering the availability of data, practical possibilities and interrelationships among the factors, the demographic factors include population growth and population ageing and the non-demographic factors include disease prevalence rate, expenditure per prevalent case and EHPI was selected in the model. Therefore, health expenditure in time *t* can be expressed as the product of these factors:

$$ {E}_t={\displaystyle \sum_{i=1}^n{\displaystyle \sum_{j=1}^m Po{p}_t\times {S}_{i jt}}\times {R}_{i jt}}\times R E\_ per\; c a s{e}_{i jt}\times {I}_{i jt} $$

where *i* represents disease group, *j* represents age group. *E*_*t*_ represents health expenditure in time *t*, *Pop*_*t*_ is the total population at time *t*, *S*_*jt*_ is the share of population of disease *i* in age group *j* at time *t, R*_*ijt*_ is the prevalence rate of disease *i* in age group *j* at time *t*, *RE_per case*_*ijt*_, is expenditure per prevalent case in constant prices (hereinafter “expenditure per prevalent case”) of disease *i* in age group *j* at time *t*, *I*_*ijt*_ is the EHPI of disease *i* in age group *j* at time *t*.

If *E*_*it*_ represents health expenditure on disease *i* at time *t* and *E*_*iT*_ represents health expenditure on disease *i* at time *T*, consequently, the difference (*E*_*d*_) of health expenditure on disease *i* at time *t* and *T* can be expressed as:

$$ \begin{array}{l}{E}_d={E}_{iT}-{E}_{it}=\mathrm{Population}\ \mathrm{growth}\ \mathrm{effect}+\mathrm{Ageing}\;\mathrm{effect} + \mathrm{Disease}\ \\ {}\mathrm{prevalence}\ \mathrm{rate}\ \mathrm{effect}+\mathrm{Expenditure}\ \mathrm{per}\ \mathrm{prevalent}\ \mathrm{case}\ \mathrm{effect}+\mathrm{EHPI}\;\mathrm{effect}\end{array} $$

Interaction effects among the five factors can be distributed across the main effects using Das Gupta’s decomposition method [19]. 32 counterfactual scenarios were developed by a Das Gupta’s decomposition Stata module to represent the principal drivers of changes in health expenditure in China, then different numbers for health expenditure using the above 32 scenarios were computed, and the net change in these scenarios is equal to the actual total change of health expenditure.

We chose 1993 as the base year for our modelling because in the 1990s health expenditure by age group and by disease group was only available for 1993 [20]. Also there was a significant policy change in 1992 when the government introduced user fees throughout the country so as to tackle inefficiencies in health service delivery and meet health demands better [21], so this made 1993 appropriate as a base year. Data for health expenditure by age group and by disease group were next available for 2012 [22], so the year 2012 was selected as the end date.

### Data sources

Population data in 1993 and 2012 were obtained from the *Population Division, Department of Economic and Social Affairs, United Nations* [23]. Population was divided into eight subgroups in this study: 0–4, 5–9, 10–19, 20–29, 30–39, 40–49, 50–59, 60+, because disease-specific health expenditure in 1993 could only be disaggregated into these eight age groups. Therefore, this analysis cannot reflect differences in the ageing of the population group 60 years and above.

Disease prevalence rate by age group and disease group was calculated using prevalent cases by age group and disease group divided by population by age group. Disease prevalent case data by age and disease in 1990, 1995, 2000, 2005, 2010 and 2013 were from the GBD 2013 Study by the *Institute for Health Metrics and Evaluation (IHME), University of Washington* [24]. ICD10 chapters were employed as the disease categories in this study. 250 causes from IHME were coded with ICD10 disease codes according to the *List of International Classification of Disease Codes Mapped to Global Disease Burden* (GBD) *Cause List* [25]. Chapter XVIII Symptoms, signs and abnormal clinical and laboratory findings, not elsewhere classified, chapter XXI Factors influencing health status and contact with health services and chapter XXII Codes for special purposes are not included, because there is no cause from IHME which can be allocated against these three chapters. Chapter XX External causes of morbidity and mortality was grouped into Chapter XIX Injury, poisoning and certain other consequences of external causes, as age- and disease-specific health expenditure data of these two chapters are grouped. Therefore, 18 ICD10 chapters were finally used in this study, which are consistent with disease categories for health expenditure in 1993 and 2012. The disease prevalence rate and the annual growth rate of the disease prevalence rate were calculated for each of these 18 ICD10 chapters. We calculated the disease prevalence rate by age and disease in 1993 and 2012 by assuming the annual growth rate of disease prevalence rate by age and disease between 1990 and 1995 was the same as the period from 1990 to 1993 and the annual growth rate of the disease prevalence rate by age and disease between 2010 and 2013 was the same as the period from 2010 to 2012.

EHPI is a measure of the amount that the change in prices within the health goods and services sector of the economy exceeds general inflation [26]. An aggregate health price index was calculated using the Laspeyres price index method [27]. The EHPI was 1.1% per year during the period 2007 to 2012. We assumed that EHPI during the period 1993 to 2012 was the same as EHPI from 2007 to 2012; this assumption was necessary as there were no comprehensive reliable data about health price increases for the period 1993 to 2012. But such data as were available indicated that EHPI was certainly positive during this period.

Expenditure per prevalent case of disease *i* in age group *j* was calculated using health expenditure of disease *i* in age group *j* divided by population of age group *j*, prevalence rate of disease *i* in age group *j* and EHPI. Therefore, the effect of expenditure per prevalent case on changes in health expenditure includes what is in other studies frequently called the residual factor. This factor includes the impact of technological progress, the expansion of health insurance and income and the impact of urbanisation, etc.

Health expenditure estimates by disease group and by age group in 1993 and 2012 were sourced from the China National Health Accounts Studies [20, 22].

### Sensitivity analysis

To examine the stability of the decomposition results, a sensitivity analysis was undertaken to gain insight into the effect in the model of the level of uncertainty with regard to disease prevalence rates. Therefore, lower and upper confidence bounds of prevalence rate by age and disease were calculated using lower and upper bound of prevalent case by age and disease (with a 95% confidence interval) divided by population by age group. The mean of disease prevalence rate by age group refers to the medium assumptions used in the model. Lower and upper confidence bounds of prevalence rate from IHME were used to perform the sensitivity analysis. Scenario 1: lower value of disease prevalence rate by age group was adopted; scenario 2: the upper value of disease prevalence rate was adopted.

## Results

**Fig. 1:**
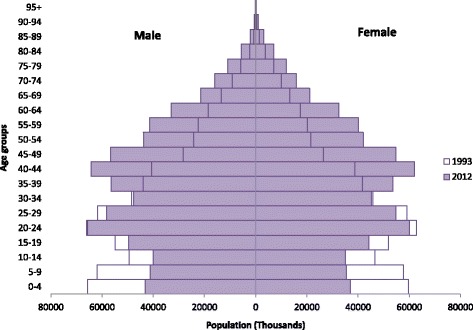
The changes in age structure in China from 1993 to 2012

During the period from 1993 to 2012, the population in China increased from 1203.0 million to 1355.4 million, and the age structure has changed significantly. The share of the age groups 0–4, 5–9, 10–19 and 20–29 decreased from 10.4%, 9.9%, 16.9% and 20.8% to 6.7%, 6.4%, 14.1% and 19.8%, respectively, while the share of the age groups 30–39, 40–49, 50–59, 60+ sharply increased from 15.0%, 11.1%, 7.4% and 8.6% to 16.9%, 19.8%, 13.9% and 15.2%, respectively. Figure 1 shows the changes in age structure in China from 1993 to 2012.

During the same period, the age-standardised disease prevalence rate decreased from 357.1% (95% CI 302.0–431.0%) to 304.0% (95% CI 262.4–358.5%). In particular, the disease prevalence rate of infectious and parasitic diseases decreased from 111.3% (95% CI 84.3–146.8%) to 64.6% (95% CI 51.4–82.0%), blood-related disease decreased from 39.8% (95% CI 38.7–41.5%) to 36.7% (95% CI 35.5–38.3%), and injury and poisoning decreased from 8.1% (95% CI 5.8–10.4%) to 6.1% (95% CI 4.5–8.0%), while the age-standardised prevalence rate of some diseases increased. For instance, the prevalence rate for skin diseases increased from 25.3% (95% CI 18.5–37.4%) to 26.6% (95% CI 18.5–38.6%), for endocrine, nutritional and metabolic diseases it increased from 7.3% (95% CI 6.1–8.4%) to 8.2% (95% CI 7.3–9.2%), for genitourinary diseases it increased from 14.7% (95% CI 11.6–17.7%) to 15.2% (95% CI 12.6–19.0%), and respiratory diseases and neoplasms also showed a slight increase. These trends are consistent with the changes in age-standardised disease prevalence rates in the National Household Health Survey Report [28].

Expenditure per prevalent case of each disease in each age group increased rapidly during the period 1993 to 2012 (Additional file 2).

**Fig. 2:**
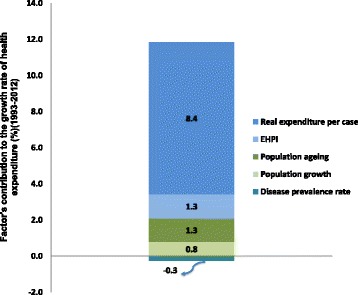
Each factor’s contribution to the growth rate of health expenditure during the period 1993 to 2012

Real health expenditure increased sharply from 124.5 billion Yuan in 1993 to 1000.6 billion Yuan in 2012 in constant 1993 prices. The real annual average growth rate was 11.6% during those two decades. The growth of health expenditure in China was mainly driven by a rapid increase in expenditure per case, while the effects of EHPI and demographic factors were small. On the other hand, a decline in disease prevalence rates led to small savings in health expenditure. Figure 2 shows each factor’s contribution to the growth rate of health expenditure during the period 1993 to 2012. 8.4% of the growth was caused by the change of real health expenditure per case, followed by EHPI, population ageing and population growth, which contributed 1.3, 1.3 and 0.8%, respectively. Changes in the disease prevalence rate contributed −0.3% of the growth in health expenditure in past two decades.

**Fig. 3:**
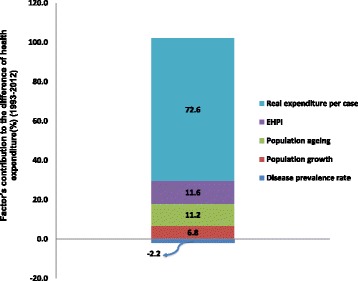
Each factor’s contribution to the difference of health expenditure during the period 1993 to 2012

Over the past two decades, health expenditure in China increased 876.1 billion Yuan in constant 1993 prices. 72.6% of the change was caused by the change in health expenditure per case, followed by EHPI, population ageing and population growth, which contributed 11.6, 11.2 and 6.8%, respectively. On the other hand, a reduction in disease prevalence rates led to savings of 2.2% (see Fig. 3).

The changes in expenditure per prevalent case mainly occurred with circulatory diseases, respiratory diseases, neoplasms, endocrine, nutritional and metabolic diseases, digestive diseases and injury and poisoning, which contributed 49.4% of the increase in China’s health expenditure.

Population ageing affected circulatory diseases the most, which caused 4.1% of the increase in health expenditure, followed by neoplasms (1.9%), endocrine and nutrition (1.3%), digestive (1.3%), musculoskeletal diseases (1.0%) and injury, poisoning and other consequences of external causes (0.9%), while ageing alleviated the growth of health expenditure on certain conditions originating in the perinatal period (−0.3%), respiratory disease (−0.2%), maternal disorders (−0.2%) and congenital malformations (−0.1%).

Population growth and EHPI increased annually 0.6% and 1.1%, respectively, in the past two decades, which mainly affected the top six diseases (circulatory, respiratory, neoplasms, endocrine, injuries and digestive diseases). The change in the prevalence rate of disease mostly reduced the growth in health expenditure. For example, changes in the prevalence rates of injuries, infectious and parasitic diseases and circulatory diseases alleviated the increase in health expenditure most, which is −1.6%, −1.4% and −0.8%, respectively. However, for some diseases the prevalence rate increased and this increased expenditure. The increase in the neoplasm prevalence rate increased expenditure by 0.8%, the increase in the prevalence rate for endocrine, nutritional and metabolic diseases increased expenditure by 0.7%, for maternal disorders expenditure increased by 0.4%, and for respiratory disease, expenditure increased by 0.2% due to the change in the prevalence rate.

Our results reflect each factor’s contribution to the change in health expenditure by disease system over the period from 1993 to 2012. Circulatory diseases, respiratory diseases, neoplasms, and endocrine, nutritional and metabolic diseases and digestive diseases caused 70.5% of the increase in current health expenditure. Each factor’s contribution to the change in health expenditure for each disease was analysed based on the result (see Table 1). For instance, circulatory disease increased by 181.6 billion Yuan during the period 1993 to 2012, which accounted for 20.7% of the increase in health expenditure. Sixty-seven percent of the increase in expenditure on cardiovascular disease was caused by expenditure per prevalent case, followed by population ageing, EHPI and population growth, which contributed 19.9, 10.7 and 6.3%, respectively, while the prevalence rate contributed −3.8%.

**Table 1:** Each factor’s contribution to the difference of health expenditure by disease during the period 1993 to 2012

Disease	Difference of health expenditure (billion Yuan, 1993 prices)	Disease prevalence rate contribution (%)	Population growth contribution (%)	Age structure change contribution (%)	Expenditure per prevalent case contribution (%)	Excess health price inflation contribution (%)	Subtotal (%)
Diseases of the circulatory system	181.61	−0.79(−3.81)	1.30(6.27)	4.13(19.93)	13.88(66.96)	2.21(10.65)	20.73(100.00)
Neoplasms	101.14	0.77(6.65)	0.69(6.02)	1.88(16.29)	7.02(60.84)	1.18(10.21)	11.54(100.00)
Diseases of the respiratory system	96.85	0.21(1.88)	0.90(8.12)	−0.19(−1.73)	8.61(77.92)	1.53(13.81)	11.06(100.00)
Endocrine, nutritional and metabolic diseases	90.49	0.69(6.68)	0.52(4.99)	1.28(12.44)	6.97(67.45)	0.87(8.44)	10.33(100.00)
Diseases of the digestive system	77.38	−0.38(−4.34)	0.74(8.35)	1.26(14.22)	5.97(67.56)	1.26(14.21)	8.85(100.00)
Injury, poisoning and certain other consequences of external causes	69.57	−1.60(−20.10)	0.63(7.89)	0.86(10.77)	6.99(88.01)	1.07(13.43)	7.95(100.00)
Diseases of the genitourinary system	66.02	0.11(1.42)	0.47(6.18)	0.69(9.15)	5.48(72.76)	0.79(10.49)	7.54(100.00)
Diseases of the musculoskeletal system and connective tissue	49.05	−0.04(−0.72)	0.38(6.81)	0.98(17.43)	3.63(64.92)	0.65(11.57)	5.60(100.00)
Pregnancy, childbirth and the puerperium	26.25	0.40(13.29)	0.19(6.41)	−0.15(−5.16)	2.23(74.58)	0.33(10.88)	3.00(100.00)
Certain infectious and parasitic diseases	24.43	−1.38(−49.59)	0.28(10.11)	0.00(0.14)	3.4(122.08)	0.48(17.25)	2.78(100.00)
Diseases of the nervous system	22.97	−0.20(−7.46)	0.18(6.91)	0.32(12.29)	2.01(76.53)	0.31(11.74)	2.62(100.00)
Mental and behavioural disorders	15.38	−0.02(−1.16)	0.11(6.12)	0.19(11.03)	1.29(73.62)	0.18(10.39)	1.75(100.00)
Diseases of the skin and subcutaneous tissue	12.59	0.01(0.55)	0.09(6.44)	0.05(3.56)	1.13(78.53)	0.16(10.93)	1.44(100.00)
Diseases of the ear and mastoid process	11.00	−0.06(−4.89)	0.07(5.59)	0.10(7.99)	1.03(81.84)	0.12(9.47)	1.26(100.00)
Diseases of the eye and adnexa	10.91	−0.08(−6.50)	0.09(7.30)	0.14(11.47)	0.94(75.32)	0.15(12.41)	1.24(100.00)
Certain conditions originating in the perinatal period	8.40	0.13(14.06)	0.06(6.12)	−0.28(−29.28)	0.95(98.73)	0.10(10.37)	0.96(100.00)
Congenital malformations, deformations and chromosomal abnormalities	6.55	0.13(17.37)	0.04(5.94)	−0.08(−10.35)	0.58(76.95)	0.08(10.08)	0.75(100.00)
Diseases of the blood and blood-forming organs and certain disorders involving the immune mechanism	5.45	−0.06(−9.30)	0.06(9.16)	0.05(7.81)	0.48(76.71)	0.10(15.62)	0.63(100.00)
Total	876.05	−2.17	6.80	11.23	72.59	11.55	100.00

*Notes*: 1. Numbers in parentheses are the factor’s contribution to the difference of expenditure on relevant disease in first column and numbers out of brackets are the factor’s contribution to the difference of total current health expenditure. 2. Components may not add to totals due to rounding

Sensitivity analysis showed the decomposition estimates of growth rate and the changes in health expenditure are robust. Table 2 illustrates the factors’ effect on the growth rate (11.59%) of health expenditure during the research period in different scenarios. 8.41–8.51% out of the overall growth of 11.59% was caused by the changes of real expenditure per prevalent case, followed by EHPI (1.339–1.343%) and population ageing (1.30–1.31%). Population growth contributed 0.788–0.791% of the growth, while changes in the disease prevalence rate reduced the overall health expenditure growth, contributing from −0.37 to −0.25% to growth.

**Table 2:** Each factor’s contribution to the growth of health expenditure in different scenarios

Drivers	Scenario 1 (%)	Medium assumption	Scenario 2 (%)
Disease prevalence rate	−0.370	−0.252	−0.312
Population growth	0.791	0.788	0.789
Population ageing	1.312	1.302	1.297
Excess health price inflation	1.343	1.339	1.342
Real expenditure per prevalent case	8.514	8.414	8.473
Total	11.591	11.591	11.591

Table 3 shows each factor’s contribution to the change in health expenditure from 1993 to 2012 in the different scenarios. Real expenditure per prevalent case contributed 72.59–73.45% of the change in health expenditure, the prevalence rate contribution ranged from −3.19 to −2.17%, EHPI contributed 11.55–11.59%, population ageing contributed 11.19–11.32% and population growth contributed 6.80–6.82%.

**Table 3:** Each factor’s contribution to the change in health expenditure in different scenarios

Drivers	Scenario 1 (%)	Medium assumption	Scenario 2 (%)
Disease prevalence rate	−3.19	−2.17	−2.69
Population growth	6.82	6.80	6.81
Population ageing	11.32	11.23	11.19
Excess health price inflation	11.59	11.55	11.58
Real expenditure per prevalent case	73.45	72.59	73.10
Total	100.00	100.00	100.00

From the results we can see that the trend of the numbers in Tables 2 and 3 is nonlinear. For example, when we used the upper confidence bound of the prevalence rate in the sensitivity analysis it did not show a greater contribution to the changes in health expenditure. This is because the changes in prevalence rate in 1993 and 2012 of the upper confidence bound are not always greater than that of the lower confidence bound or the mean prevalence rate. For instance, during the research period, the upper confidence bound of the prevalence rate of population aged 50–59 with endocrine, nutritional and metabolic diseases increased by 3.50% (from 15.64 to 19.14%), which is higher than the change of the mean prevalence rate of population aged 50–59 with this kind of disease (3.48%, increased from 13.41 to 16.90%), although less than the change of the lower confidence bound of the prevalence rate of population aged 50–59 with this kind of disease (3.55%, increased from 11.19 to 14.73%).

## Discussion

Compared with regression based methods, Das Gupta’s decomposition method can fully decompose the growth of disease-specific health expenditure between two time points into selected effecting factors, and with full allocation of interaction effects. Major decomposition results in this study from Das Gupta’s method are consistent with views from empirical studies.

For example, empirical evidence suggests that the age structure of the population has only a modest impact on the growth of health expenditure [29]. Similarly, this study showed that population ageing was not a significant driver of health expenditure growth in China. In addition, longevity gains will progressively postpone health expenditure from one age class to the next, reducing further the impact of population ageing [30]. However, the proportion of population aged 60+ in China is projected to be 25.4% in 2030 even under the two-child policy implemented in 2016, therefore, proportion of the growth of health expenditure in the future will increase due to population ageing.

Expenditure per prevalent case was the main factor that drove the growth of health expenditure over this period. Growth in expenditure per prevalent case is in turn driven by factors such as income growth, technological advances, health insurance changes, consumer preferences and changes in service provision practices by health providers. Further work could analyse the exact role of these factors in driving growth in expenditure per prevalent case. Another driver of increases in expenditure per prevalent case is providers’ profit seeking behaviour. The government sets prices for simple and non-invasive services well below cost, while setting prices above cost for high-tech diagnostics and examinations [31]. Furthermore, the government allows a 15% profit margin on pharmaceuticals. This price schedule has created perverse incentives for providers, who have to generate 90% of their budget from revenue-generation [21], which causes increases in charges per outpatient visit and inpatient admission. During the period 1993 to 2012, outpatient charges per capita increased from 29.1 Yuan (US$ 3.5) to 80.2 Yuan (US$ 12.7) and per capita inpatient charges increased from 1216.4 Yuan (US$ 145.7) to 2992.3 Yuan (US$ 474.0) in constant 1993 prices [32]. Additionally, patients tend to utilise health services with higher price in higher level hospitals in the past two decades. For example, the proportion of total visits in township hospitals decreased from 59.1% in 1993 to 28.8% in 2012, and proportion of total discharges provided in township hospitals went down from 48.7 to 26.1% during the same period [32], due to rapid urbanisation and low provision capacity of grass-root health facilities.

The other major driver was EHPI. The health care sector has a higher proportion of high-skilled workers than average, and salary levels for high-skilled workers rise faster than for low-skilled workers in the labour market [33]. In China, the number of people with a junior college degree increased from 5.6% in 2005 to 10.6% in 2012, while among health personnel, the number increased from 46.3 to 64.3% during the same period [34]. Higher health price inflation is not unique to China. For example, EHPI in the United Kingdom was 1.6% per year [33]. Health service pricing reform is one of the main objectives of the new round of health system reform being undertaken in China, with the aim to adjust prices so they better reflect resource costs. However, research has revealed that the regulated health service price may actually undermine equitable access to care [31].

The decrease in the prevalence rate of injuries and poisoning, and infectious and parasitic diseases slowed down the growth of health expenditure. That is partly because a series of regulations and laws related to safety in production and transport, occupational injuries and poisoning have been issued by the government to prevent and control the prevalence of injury [35]. Furthermore, China has made great progress in infectious disease control. Through establishing a disease control and prevention system, promoting vaccination and organising a patriotic health campaign, China has successfully lowered the incidence of infectious diseases and effectively controlled most major infectious diseases [36]. On the other hand, China now faces emerging challenges in the increasing burden of some non-communicable diseases (NCDs) and the rising prevalence of some risk factors. NCDs are responsible for 77% of the loss in healthy life and 85% of all deaths, a profile similar to that of most Organisation for Economic Co-operation and Development (OECD) countries [37]. High-risk behaviours such as smoking, poor diets, sedentary lifestyles and alcohol consumption, as well as environmental factors such as air pollution, are powerful forces behind the emergence of chronic illnesses in China [38].

## Conclusion

In conclusion, we have decomposed the growth of health expenditure over a 20 year period in China. We found that expenditure per prevalent case was the major driver of health expenditure growth, and the effect of economic growth and technological progress may be absorbed in the effect of expenditure per prevalent case. EHPI played a secondary role, and demographic factors were a small contributor to growth, while reductions in disease prevalence rate slowed down the growth to some extent. Moreover, we find that the growth of health expenditure for neoplasms, and circulatory, respiratory, endocrine, nutritional and metabolic and digestive diseases over the period dramatically drove the increase in health expenditure. Our results indicate that population ageing played a more important role in the growth of circulatory, neoplasms, and endocrine, nutritional and metabolic, digestive and musculoskeletal diseases expenditure than other disease expenditures.

Our work highlights that reforms which constrain growth in health expenditure per case of disease and EHPI, especially for neoplasms and circulatory, respiratory, endocrine, nutritional and metabolic and digestive diseases, are fundamental to the control of the rapid growth of health expenditure in China. Enlarging the service package of the Program for the Universal Coverage of Essential Public Health Services implemented from 2009 may be a desirable option to further reduce the disease prevalence rate. Strengthening the capacity of health personnel in grass-roots facilities and establishing an effective referral system can help to moderate the rapid growth of expenditure per prevalent case and ensure that extra services are provided in areas where they will achieve the greatest health gain. EHPI needs to be controlled to ensure that extra resources result in extra services rather than higher incomes for health providers. Further research into region-specific drivers of health expenditure growth will enable expenditure optimising measures to be better targeted while at the same time achieving equity goals.

## Additional files

Additional file 1: Das Gupta’s decomposition equation. A brief introduction of the Das Gupta’s decomposition equation. (DOCX 21 kb)
Additional file 2: Expenditure per prevalent case by age group and disease in 1993 and 2012, China. This dataset illustrates the changes of expenditure per prevalent case by age group and disease during the period 1993 and 2012 in China constant in 1993 prices. (DOCX 41 kb)

## Abbreviations

EHPI: Excess health price inflation
GDP: Gross domestic product
ICD: International classification of diseases
IHME: Institute for Health Metrics and Evaluation
NCDs: Non-communicable diseases
OECD: Organisation for Economic Co-operation and Development.
