# Supplementary material for: Main drivers of health expenditure growth in China: a decomposition analysis
Source: BMC Health Serv Res. 2017 Mar 9;17:185. doi: 10.1186/s12913-017-2119-1 (PMC5343399; doi:10.1186/s12913-017-2119-1)
Supplement: Additional file 1: — Das Gupta’s decomposition equation. A brief introduction of the Das Gupta’s decomposition equation. (DOCX 21 kb) [file 12913_2017_2119_MOESM1_ESM.docx]

**Appendix 1 Das Gupta’s decomposition equation**

Prithwis Das Gupta’s decomposition method can be used in two situations: an index can be expressed as a function of two or three factors and the data involve one or more factors organized in cross-classified style. We adapted the former: an index can be expressed as the product of several factors. For example, when the index is the product of five factors α, β, γ, μ and δ, if these factors assume the values A, B, C, D and E in group 1, and a, b, c, d and e in group2, then the indexes I_1_ and I_2_ in two groups are: I_1_ = ABCDE, I_2_ = abcde. The α effect in this case is given by

$$\alpha effect=[\frac{bcde+BCDE}{5}+\frac{\begin{aligned} b\mathrm{cd}E+bcDe+bCde+Bcde \\ + BCDe+BCdE+BcDE+bCDE \end{aligned}}{20}+\frac{\begin{aligned} bcDE+bCdE+bCDe \\ +BCde+BcDe+BcdE \end{aligned}}{30}](a-A)$$

The expressions for other effects can be derived easily from above equation for α effect. For example, the β effect is obtained from α effect by substituting b, a, B and A for a, b, A and B, respectively.

I_2_ - I_1_ = α effect + β effect + γ effect + μ effect + δ effect

**Reference**

1. Gupta PD. Decomposition of the difference between two rates and its consistency when more than two populations are involved. *Mathematical Population Studies* 1991; **3**: 105–25.
2. Gupta PD. Standardization and decomposition of rates: A user's manual. US Department of Commerce, Economics and Statistics Administration, Bureau of the Census, 1993.
