# Supplementary material for: Main drivers of health expenditure growth in China: a decomposition analysis
Source: BMC Health Serv Res. 2017 Mar 9;17:185. doi: 10.1186/s12913-017-2119-1 (PMC5343399; doi:10.1186/s12913-017-2119-1)
Supplement: Additional file 2: — Expenditure per prevalent case by age group and disease in 1993 and 2012, China. This dataset illustrates the changes of expenditure per prevalent case by age group and disease during the period 1993 and 2012 in China constant in 1993 prices. (DOCX 41 kb) [file 12913_2017_2119_MOESM2_ESM.docx]

**Appendix 2 Expenditure per prevalent case by age group and disease in 1993 and 2012, China**

| **Disease** | **Age group** | **1993 (Yuan)** | **2012 (Yuan, constant in 1993 prices)** |
| --- | --- | --- | --- |
| Certain infectious and parasitic diseases | 0～4 | 13.9 | 173.9 |
|  | 5～9 | 7.4 | 28.1 |
|  | 10～19 | 4.1 | 10.7 |
|  | 20～29 | 4.5 | 22.6 |
|  | 30～39 | 5.7 | 25.4 |
|  | 40～49 | 4.1 | 22.5 |
|  | 50～59 | 5.8 | 27.9 |
|  | 60＋ | 4.1 | 30.9 |
| Neoplasms | 0～4 | 168.2 | 4784.2 |
|  | 5～9 | 2339.6 | 11656.1 |
|  | 10～19 | 2061.3 | 6223.1 |
|  | 20～29 | 125.7 | 1395.7 |
|  | 30～39 | 179.7 | 840.9 |
|  | 40～49 | 172.0 | 754.7 |
|  | 50～59 | 607.8 | 2830.0 |
|  | 60＋ | 1388.8 | 5565.2 |
| Diseases of the blood and blood-forming organs and certain disorders involving the immune mechanism | 0～4 | 0.6 | 7.3 |
|  | 5～9 | 0.6 | 10.2 |
|  | 10～19 | 1.0 | 9.0 |
|  | 20～29 | 3.6 | 8.0 |
|  | 30～39 | 6.6 | 9.0 |
|  | 40～49 | 4.5 | 12.2 |
|  | 50～59 | 10.1 | 17.8 |
|  | 60＋ | 7.7 | 32.5 |
| Endocrine, nutritional and metabolic diseases | 0～4 | 30.2 | 1070.4 |
|  | 5～9 | 3.9 | 321.7 |
|  | 10～19 | 7.8 | 163.6 |
|  | 20～29 | 20.4 | 222.2 |
|  | 30～39 | 26.6 | 279.3 |
|  | 40～49 | 20.2 | 384.9 |
|  | 50～59 | 68.5 | 706.4 |
|  | 60＋ | 47.1 | 912.2 |
| Mental and behavioural disorders | 0～4 | 2.5 | 258.0 |
|  | 5～9 | 1.3 | 29.9 |
|  | 10～19 | 3.3 | 19.5 |
|  | 20～29 | 6.4 | 45.1 |
|  | 30～39 | 11.0 | 68.5 |
|  | 40～49 | 11.8 | 76.7 |
|  | 50～59 | 12.4 | 101.3 |
|  | 60＋ | 15.7 | 100.4 |
| Diseases of the nervous system | 0～4 | 67.9 | 5398.6 |
|  | 5～9 | 7.1 | 349.1 |
|  | 10～19 | 4.9 | 37.3 |
|  | 20～29 | 7.7 | 22.4 |
|  | 30～39 | 12.2 | 27.9 |
|  | 40～49 | 15.1 | 42.1 |
|  | 50～59 | 19.4 | 92.5 |
|  | 60＋ | 22.9 | 193.7 |
| Diseases of the eye and adnexa | 0～4 | 7.4 | 163.5 |
|  | 5～9 | 6.7 | 108.8 |
|  | 10～19 | 15.7 | 67.8 |
|  | 20～29 | 18.8 | 82.8 |
|  | 30～39 | 30.7 | 81.3 |
|  | 40～49 | 21.7 | 59.7 |
|  | 50～59 | 19.6 | 62.3 |
|  | 60＋ | 17.6 | 87.9 |
| Diseases of the ear and mastoid process | 0～4 | 9.4 | 141.2 |
|  | 5～9 | 5.1 | 66.9 |
|  | 10～19 | 5.5 | 44.0 |
|  | 20～29 | 2.6 | 107.4 |
|  | 30～39 | 2.9 | 78.8 |
|  | 40～49 | 3.1 | 63.1 |
|  | 50～59 | 1.1 | 20.7 |
|  | 60＋ | 0.7 | 10.0 |
| Diseases of the circulatory system | 0～4 | 47.3 | 1604.6 |
|  | 5～9 | 33.0 | 777.1 |
|  | 10～19 | 52.0 | 513.5 |
|  | 20～29 | 142.8 | 890.3 |
|  | 30～39 | 415.1 | 1754.7 |
|  | 40～49 | 278.8 | 1458.3 |
|  | 50～59 | 541.0 | 1909.4 |
|  | 60＋ | 470.1 | 2599.7 |
| Diseases of the respiratory system | 0～4 | 1742.2 | 14170.3 |
|  | 5～9 | 720.3 | 4492.9 |
|  | 10～19 | 377.5 | 994.2 |
|  | 20～29 | 316.0 | 787.1 |
|  | 30～39 | 318.8 | 737.9 |
|  | 40～49 | 246.7 | 611.5 |
|  | 50～59 | 254.4 | 606.1 |
|  | 60＋ | 253.7 | 1042.2 |
| Diseases of the digestive system | 0～4 | 23.9 | 110.7 |
|  | 5～9 | 5.4 | 30.1 |
|  | 10～19 | 8.4 | 37.9 |
|  | 20～29 | 23.2 | 58.8 |
|  | 30～39 | 39.4 | 81.5 |
|  | 40～49 | 46.1 | 113.6 |
|  | 50～59 | 56.6 | 145.1 |
|  | 60＋ | 43.6 | 173.7 |
| Diseases of the skin and subcutaneous tissue | 0～4 | 55.4 | 343.5 |
|  | 5～9 | 10.5 | 57.3 |
|  | 10～19 | 2.4 | 14.2 |
|  | 20～29 | 2.2 | 19.1 |
|  | 30～39 | 4.9 | 34.3 |
|  | 40～49 | 6.6 | 39.6 |
|  | 50～59 | 6.5 | 40.6 |
|  | 60＋ | 4.8 | 46.3 |
| Diseases of the musculoskeletal system and connective tissue | 0～4 | 210.0 | 3669.7 |
|  | 5～9 | 98.8 | 1064.8 |
|  | 10～19 | 14.9 | 141.7 |
|  | 20～29 | 15.2 | 96.3 |
|  | 30～39 | 30.1 | 109.9 |
|  | 40～49 | 39.5 | 131.0 |
|  | 50～59 | 45.9 | 154.4 |
|  | 60＋ | 35.2 | 141.5 |
| Diseases of the genitourinary system | 0～4 | 19.4 | 566.5 |
|  | 5～9 | 38.3 | 313.5 |
|  | 10～19 | 23.3 | 146.6 |
|  | 20～29 | 25.1 | 212.8 |
|  | 30～39 | 34.5 | 246.8 |
|  | 40～49 | 49.0 | 219.6 |
|  | 50～59 | 43.9 | 269.8 |
|  | 60＋ | 44.7 | 294.2 |
| Pregnancy, childbirth and the puerperium | 0～4 | — | — |
|  | 5～9 | — | — |
|  | 10～19 | 22.0 | 409.7 |
|  | 20～29 | 346.4 | 1716.4 |
|  | 30～39 | 95.4 | 1289.4 |
|  | 40～49 | 107.2 | 1115.1 |
|  | 50～59 | — | — |
|  | 60＋ | — | — |
| Certain conditions originating in the perinatal period | 0～4 | 96.0 | 4720.9 |
|  | 5～9 | 13.5 | 685.2 |
|  | 10～19 | 2.0 | 76.1 |
|  | 20～29 | 5.7 | 194.7 |
|  | 30～39 | 1.3 | 37.4 |
|  | 40～49 | 1.0 | 18.5 |
|  | 50～59 | 1.4 | 24.1 |
|  | 60＋ | 3.8 | 84.4 |
| Congenital malformations, deformations and chromosomal abnormalities | 0～4 | 121.3 | 1498.6 |
|  | 5～9 | 71.8 | 566.4 |
|  | 10～19 | 15.6 | 304.2 |
|  | 20～29 | 7.9 | 203.4 |
|  | 30～39 | 42.0 | 197.8 |
|  | 40～49 | 32.1 | 225.2 |
|  | 50～59 | 44.7 | 266.4 |
|  | 60＋ | 53.3 | 265.1 |
| Injury, poisoning and certain other consequences of external causes | 0～4 | 523.7 | 3951.7 |
|  | 5～9 | 362.4 | 1571.8 |
|  | 10～19 | 187.3 | 906.2 |
|  | 20～29 | 173.2 | 898.6 |
|  | 30～39 | 146.4 | 704.2 |
|  | 40～49 | 136.0 | 690.9 |
|  | 50～59 | 123.0 | 523.2 |
|  | 60＋ | 77.0 | 452.9 |

**Source:** Calculated by authors.
